# Treating the musician rather than the symptom: The holistic tools employed by current practices to attend to the non-motor problems of musicians with task-specific focal dystonia

**DOI:** 10.3389/fpsyg.2022.1038775

**Published:** 2023-01-13

**Authors:** Anna Détári

**Affiliations:** School of Arts and Creative Technologies University of York, York, United Kingdom

**Keywords:** Musician’s Focal Dystonia, treatment, holistic approach, ongoing practices, interview with practitioners

## Abstract

Musicians Focal Dystonia (MFD) is a task-specific movement disorder affecting highly skilled musicians. The pathophysiology is poorly understood, and the available treatments are unable to fully and reliably rehabilitate the affected skill. Recently, the exclusively neurological nature of the condition has been questioned, and additional psychological, behavioral, and psychosocial contributing factors were identified. However, very little is known about how these factors influence the recovery process, and how, if at all, they are addressed in ongoing practices. For this study, 14 practitioners with substantial experience in working with musicians with MFD were interviewed about the elements in their approach which are directed at the cognition, emotions, attitudes, and behaviors of their patients and clients. A wide variety of tools were reported in three areas: (1) creating a supportive learning environment and addressing anxiety and perfectionism, (2) using body-oriented methods to optimize the playing behaviors and (3) consciously channeling the focus of attention to guide the physical retraining exercises and establishing new habits. The study also revealed that in-depth knowledge of the instrumental technique is profitable to retrain the impaired motor patterns. Therefore, the importance of including music educators in developing new therapeutic approaches will also be highlighted.

## Introduction

According to the most recent estimates, 1–2% of accomplished musicians experience severe impairment in their fine motor skills when playing their instrument due to Musician’s Focal Dystonia (MFD) at some point in their careers (Altenmüller, 2003). This peculiar condition is classified as a Focal Isolated Dystonia (FID; previously Primary Focal Dystonia; Albanese et al., 2013), it is neurological in origin (Altenmüller and Jabusch, 2010), most often painless, and highly task-specific: for the majority of the affected musicians, the symptoms only appear when they attempt to perform the triggering task, i.e., playing their instrument (Hofmann et al., 2015). These symptoms can be described as tension, spasms, tremors, or other involuntary movements, and result in the loss of fine motor control over the task-specific movements (Jabusch and Altenmüller, 2006; Altenmüller et al., 2014). Most commonly, the upper extremity (fingers, wrists, forearms; Byl, 2006; Altenmüller and Jabusch, 2009), or the facial muscles, including the tongue and jaw (i.e., embouchure), are affected (Frucht et al., 2001; Termsarasab and Frucht, 2016), but some studies describe cases of lower extremity dystonia affecting drummers (Lee and Altenmüller, 2014). The onset of MFD typically appears after several years of diligent practice, most often in the prime of the musician’s career (Conti et al., 2008), and in most cases, diminishes or ends the career of the musician, and has further, severely negative financial, psychosocial, and psychological impact on the individual’s life.

The understanding of the pathophysiology of MFD and similar task-specific focal dystonias went through significant changes since the first medical descriptions in the last century (Munts and Koehler, 2010) and it is still limited (Jankovic and Ashoori, 2008; Altenmüller et al., 2014). First, it was treated as “hysteria,” an exclusively psychological condition but with the advancement of brain imaging technologies and neuroscience, this theory was heavily criticized, and the condition was reclassified as neurological (Albanese, 2017). As a result, the accompanying psychological distress was treated as a secondary reaction to the onset itself and was neglected in subsequent research (Sadnicka et al., 2016). The currently widely accepted medical models of the disorder originate the onset from the combination of genetic predisposition (Lohmann et al., 2014), impaired inhibitory processes (Byl et al., 2000; Haslinger et al., 2010), and maladaptive neuroplasticity (Byl et al., 1996; Candia et al., 2005). Various elements of these models, however, have been criticized (Sadnicka et al., 2016; Stein et al., 2016), especially the fact that they cannot explain the task-specific nature of MFD, i.e., the absence of the symptoms when the triggering task is not performed (Sadnicka et al., 2016).

Despite these criticisms, the presently used rehabilitation strategies are informed by this classification, targeting either the affected neurocircuits of the musicians by administering oral medications (Jabusch and Altenmüller, 2006) or non-invasive (Buttkus et al., 2010) and invasive brain stimulation (Horisawa et al., 2019), or symptomatically reducing the muscular symptoms of the disorder with Botulinum Toxin injections (Schuele et al., 2005; Jabusch and Altenmüller, 2006). The outcome of these therapeutic approaches is not satisfactory: while some improvement in the movement control of the affected body part is often observed, none of them can fully and reliably rehabilitate the lost skill (Sadnicka et al., 2016). More recently, the practice of somatic rehabilitation programs emerged, observing the effects of various protocols, such as constraint-induces therapies (Candia et al., 1999; Berque et al., 2010), neuromuscular re-education programs (for a review of the literature, see Enke and Poskey, 2018), slow practice (Sakai, 2006), and postural corrections (Tubiana, 2003; Ackermann and Altenmüller, 2021), or the combination of these. These therapies help the musicians to improve the impacted skill through targeted movement exercises and while they appear to be more successful than purely medical approaches, the efficiency is still moderate (Enke and Poskey, 2018).

These unsatisfactory outcomes might stem from the limited understanding of the pathophysiology and the incomplete models informing the therapeutic strategies. This prompted researchers to broaden the area of inquiry by revisiting the previously excluded psychological characteristics as risk factors and found that musicians with MFD tend to have higher levels of maladaptive perfectionism, anxiety, neuroticism, and phobias than musicians with chronic pain and healthy controls (Jabusch et al., 2004; Jabusch and Altenmüller, 2004; Enders et al., 2011). Based on retrospective inquiry and analysis, the researchers also concluded that these characteristics appear to have been present before the symptoms appeared, rather than being psychoreactive traits in response to the onset (Jabusch et al., 2004; Enders et al., 2011). Moreover, certain cognitive strategies, such as reinvestment and over-focusing on the task were also proposed as contributors to the onset (Altenmüller et al., 2014), alongside chronic overwork, playing in a fatigued state (Grünewald, 2007; Altenmüller et al., 2014; Stein et al., 2016), ill-fitted playing and practice strategies (Sadnicka et al., 2018), and trauma (Schneider et al., 2021; Alpheis et al., 2022).

A string of studies (Détári et al., 2022; Détári and Egermann, 2022a,b) also highlighted the role of the social environment in the development of the condition. Here, it was argued that the aforementioned psychological characteristics, cognitive interference, and maladaptive practice and playing strategies do not develop in a vacuum but in a social context, i.e., how the skill was learned in the first place and the characteristics of the learning and working environments have a significant role in the development of the disorder. The findings of these studies show that musicians who acquire MFD later in life often studied in a negative, sometimes even abusive environment, received little or no information about healthy playing technique and posture, and struggled with technical difficulties when playing their instruments for many years before the onset (Détári et al., 2022; Détári and Egermann, 2022b). Additionally, their heightened level of self-directed perfectionism might stem from being exposed to socially prescribed perfectionism in their homes and working and learning environments, and many musicians with MFD were actively taught to internally focus on small technical aspects of their instrumental playing rather than musicality or expressivity (Détári et al., 2022; Détári and Egermann, 2022b). These experiences and pedagogical strategies had a long-lasting influence: they informed not only the musicians’ playing technique and practice strategies but often the accompanying psychological and emotive states as well (Détári et al., 2022). It is also reasonable to suggest that these characteristics—and their impact on the playing mechanisms—do not cease to exist after the onset of the disorder, on the contrary, the trauma of losing one’s skill might even aggravate some of the existing problems, interfering with the musicians’ effective and successful participation in the treatment.

Despite the accumulated knowledge of these elements and the initiative to include them in the multifactorial etiology of the condition, there are no guidelines or protocols in the literature about how to address them during the treatment. Apart from a few unspecified pieces of advice (see Chamagne, 2003; Tubiana, 2003) regarding addressing the psychological distress of musicians with MFD, the therapeutic approaches described in the literature focus exclusively on the motor symptom. This narrow focus neglects the fact that the motor output can significantly be altered by the psychological state the person is in (Larsson et al., 1995; van Loon et al., 2001; Laursen et al., 2002), the cognitive patterns accompanying the practice (Zachry et al., 2005; Lohse et al., 2010, 2011; Wulf, 2013; Wulf and Lewthwaite, 2016), including attentional focus (Masters and Maxwell, 2008; Wulf and Lewthwaite, 2016) and previous experiences with the activity (Fuchs, 2003; Juhan, 2003; Jansen, 2012). Therefore, understanding how these additional contributing factors impact the rehabilitation of the lost skill and exploring how these influences can be reduced seems imperative to enhance the currently available therapeutic approaches.

Operating under the assumption that the lack of literature does not necessarily mean a lack of practice, the goal of this research was to explore the rehabilitation strategies of various practitioners, particularly regarding the tools they use to enhance their patients’/clients’ mindsets, psychological and emotive states, attitudes, and habitual behaviors. It was hypothesized that these previously under-researched elements of the etiology can negatively influence the process of rehabilitation, but they can be reduced by using appropriate tools to enhance the outcome of the therapy. To explore this uncharted area, interviews were conducted with practitioners who have substantial experience in treating musicians with dystonia. Opposing previous literature which reports primarily on the practice of medical professionals (e.g., neurologists, physiotherapists), in the present data collection, musician-coaches who recovered from the condition and offer therapeutic instrumental lessons to fellow musicians with the disorder were also invited to the research. These practices are largely undocumented but are highly popular among musicians with MFD (Détári et al., 2022; Détári and Egermann, 2022b) and their inclusion seemed necessary in order to gain a full picture of the support available for musicians with the disorder. Moreover, the expertise of this particular group of people is highly valuable for three reasons. Firstly, they have first-hand experience with a successful recovery process. Secondly, they have specialized instrumental expertise and extensive knowledge about correct and healthy technique, and thirdly, they have in-depth, personal experience and understanding of the challenges and stressors associated with the profession. These elements suggest a unique viewpoint and strategies differing from the medical approaches.

The participants in this research were first asked to identify the characteristics of their patients/clients and the resulting barriers to recovery before describing their strategies to address these. Due to the large amount of qualitative data and the two distinct topics of the inquiry, the decision was made to publish the findings in two separate articles. The preceding publication reports on the observations and subjective opinions of the practitioners about the personality, behaviors, attitudes, and life stories of musicians with MFD (Détári and Egermann, 2022b), while the present study focuses on the rehabilitation strategies of the participants.

Our research questions were the following:

How do psychological characteristics, emotional states, and habitual behaviors influence the outcome of the rehabilitation from MFD?

What steps are taken in ongoing practices to mitigate the influence of these?

## Materials and methods

### Participants

Participants were invited to contribute to the research based on their expertise and experience in treating musicians with MFD. Both medical professionals (e.g., neurologists, physiotherapists) and musician-coaches were asked to participate. Detailed information about the participants is presented in Table 1.

**Table 1:** The participants’ expertise and experience.

No.	Profession	Years of experience at the time of the interview
1.	Professional musician, musician-coach	11
2.	Physiotherapist (with a background in music performance)	15
3.	Professional musician, musician-coach	30
4.	Medical doctor	28
5.	Professional musician, musician-coach	15
6.	Neurologist (with a background in music performance)	19
7.	Professional musician, musician-coach	21
8.	Physiotherapist	25
9.	Researcher, medical doctor (with a background in music performance)	10
10.	Neurologist (with a background in music performance)	33
11.	Researcher, neuroscientist (with a background in music performance)	9
12.	Neurologist (with a background in music performance)	10
13.	Professional musician, musician-coach	6
14.	Professional musician, musician-coach	18

Medical professionals and researchers were selected based on their research output in peer-reviewed journals and books and their professional interactions with MFD patients. The eight participants in this group interacted with musicians with MFD in various ways, providing diagnoses, medical treatment, or physiotherapy in their practices, while two of them primarily conducted empirical studies, gathering data on the characteristics of the condition and the outcome of various treatment strategies. Six of these medical professionals were practicing musicians themselves, three had personal experience with MFD, and they had 9–33 years of experience in providing treatment for musicians with MFD.

Musician-coaches were identified *via* a thorough internet search using a combination of keywords such as “musician’s focal dystonia,” “musician’s cramp,” “therapy,” “treatment,” and “coaching.” Also, a request was placed in online support groups, asking musicians with the MFD to make recommendations of musician-coaches they have worked with or heard of, and the “snowballing” technique of qualitative inquiry (Noy, 2008), i.e., asking participants to suggest others working on the field, was also employed. Some difficulties arose with enlisting the participants in the research: six of the contacted private practitioners either ignored the request completely or refused to talk about their methods. Some of those who responded but did not wish to take part in the research expressed their concern that they would be challenged to prove that their methods work despite the clearly communicated objectives of the research. Others wished to keep their approach secret to avoid replication and “protect” their method and one practitioner stated that unless they are invited as a co-author on the resulting paper, they will not share any information. In the end, six musician-coaches agreed to take part, all of whom were professional musicians, recovered from MFD by various strategies, and had 6–30 years of experience in coaching musicians with the condition.

The combined number of participants was 14. They all work with patients/clients internationally, but their in-person practices are based in 9 different countries across the world. They had 6–33 years of experience at the time of the data collection during which they worked with approximately 2000 musicians with MFD, testing and refining their treatment strategies with a very significant pool of patients. The interviews were conducted in English.

### Procedure and analysis

Fourteen, 60–90 min long semi-structured interviews were conducted either in person or online with the participants. The interview schedule (see Appendix A) was based on the findings of the author’s previously conducted Grounded Theory study (Détári et al., 2022), and inquired about the personal characteristics and life stories of the participants’ patients and clients, the observed behaviors and non-motor symptoms and how these were addressed in the participants’ practices. As mentioned, the first part of the data about the personal characteristics of the patients/clients of the participants is reported in a separate publication (Détári and Egermann, 2022b) while the present study focuses exclusively on the rehabilitation strategies.

The analysis of the content of the interviews was data-driven, given that there were no previously established frameworks or concepts regarding the strategies employed to support the non-motor symptoms of musicians with MFD. Following the recommendations of Brinkmann and Kvale (2018), the process started with systematically identifying similarities within and across the transcribed interview texts, then establishing the main concepts, and arranging the topics into larger groups of themes to provide a clear summary of the content of the conversations. Ethical approval was granted by the Arts and Humanities Ethics Committee of the University of York and all participants were required to sign a consent form before taking part.

## Results

The participants’ practices differed greatly in terms of the quantity and frequency of the time spent with their patients and clients. Due to the nature of the administered therapy, musician-coaches, physiotherapists, and practitioners delivering behavioral interventions met with the musicians periodically, typically for sessions spanning from 30 min to 2 h. Neurologists, following the initial consultation and the prescription of the treatment, had less contact with their patients, similarly to the two researchers in the sample, who worked with their musician participants for designated periods allocated for data collection. Naturally, these differences expressed themselves in the quantity and quality of information the participants could provide on the topic: those who were in more frequent contact presented a wider variety of strategies to support the affected musicians. Nevertheless, there was a general agreement among the participants about the necessity and nature of these tools and—apart from the topic of attentional focus—similar strategies were shared during the interviews.

It became clear from preceding studies (Détári et al., 2022; Détári and Egermann, 2022b) that musicians who are affected by MFD and enter a rehabilitation program, potentially have multiple problems apart from the physical symptoms of the disorder. Some of these stems from previous traumatic experiences, some can be linked to psychological characteristics, and some are established unhealthy behaviors. These pre-existing issues are potentially aggravated by the traumatic experience of the onset, moreover, the inability to play to the accustomed level can result in financial insecurity, identity crisis, fear of the future, and for some, depression. This situation creates feelings of anger, guilt, victim identity, and lowers the musicians’ self-efficacy and self-esteem, and possibly disrupts the social network of the individual. All participants agreed that this vulnerable and complex situation can greatly influence the outcome of the rehabilitation program, and they shared various strategies which they use to address them.

**Figure 1:**
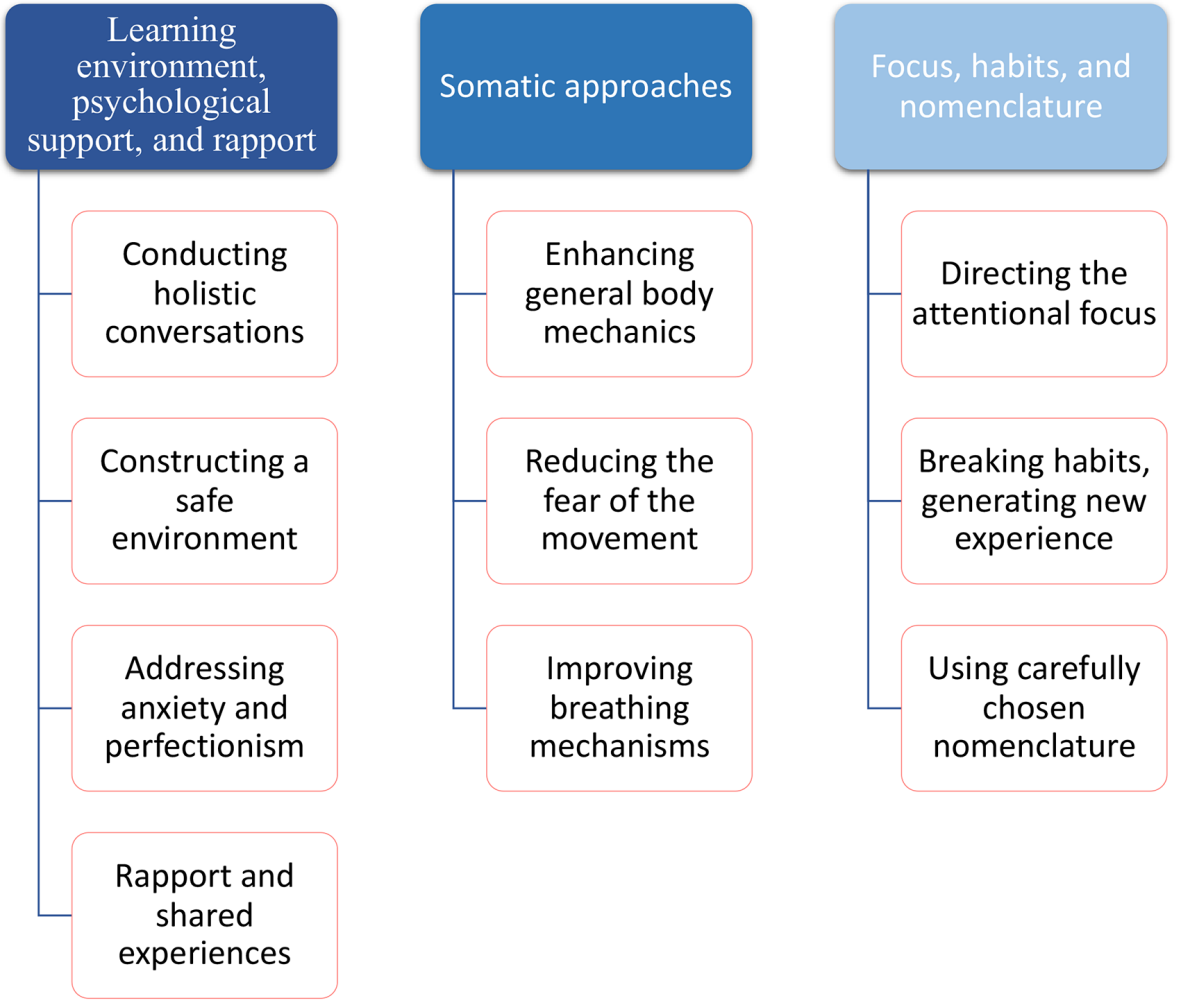
Strategies for addressing non-motor symptoms.

The findings were grouped into three categories which will be presented in the following order: (1) Learning environment, psychological support, and rapport (2) Somatic approaches (3) Focus, habits, and nomenclature. The summary of the findings is shown in Figure 1.

### Learning environment, psychological support, and rapport

#### Conducting holistic conversations

In most practices, including the patient’s experiences, feelings, and behaviors in the initial discussion is viewed as an important step. The goal of this consultation is to gather information about the specific case, but also, to make the client feel heard, understood, and accepted. Actively building rapport and finding a connection are tools frequently used by many participants; as participant No. 6. articulated: “the crucial thing is to understand the situation of the patient. The entire situation. Not only see his finger, or which finger moves in which direction.” This caring and compassionate therapeutic relationship can create a better atmosphere but also has a direct influence on the learning process of the musician: “I’m always interested in finding the right access to the person, also psychologically, so maybe they can listen more correctly to what I’m saying” (P. No. 5). One participant (P. No. 3.) even uses systematic data collection which is continuously updated when they gain new insight.

So, I have people self-report: are they right-handed or left-handed, how well can they focus their attention, how susceptible are they to depression? How self-critical are they? So, they answer a questionnaire. […] And I walk through some of that in the initial consultation as well. And I want to know if I’m coaching anybody […] are you an endurance athlete or are you a sprinter? Do you focus better in the morning? What is your schedule? What is your support system like? All of that.

These conversations also form a substantial part of the following treatment sessions to support the rehabilitation. As one practitioner (P. No. 6.) described this process:

It definitely belongs to the retraining procedure, to listen to the people and discuss… sometimes half of the session is discussion…and we go to the piano afterwards, and sometimes they feel also that they need to talk… I don’t know if I have some sort of psychologist role, but I sit down and listen. Basically, what they have to tell me. And then after, we can work correctly. But if I skip that, I think that wouldn’t be suitable for the efficiency of what we’re doing at the instrument.

#### Constructing a safe environment

Behavioral therapies are in many ways similar to traditional instrumental lessons. They are repeated frequently and exercises are introduced that the client/patient needs to practice which are monitored and evaluated in the subsequent lesson to determine the improvement. This similarity seems to evoke the participants’ original behavioral patterns: they want to please, they are afraid to make mistakes, and in some cases, they are even ashamed of their symptoms. As a musician-coach (P. No. 3.) explains: “When I first start to work with a client, I would get them to do an exercise and they do not want to, because if it is not perfect, they just stop.”

Many participants felt that changing this atmosphere was crucial for working with the patient efficiently and they defined “safety” as the most important characteristic of the learning environment. Creating such an environment happens both verbally and behaviorally; the musicians are assured that there are no expectations toward them and are treated with understanding and patience:

I’m extremely committed to the fact that I have absolutely no single expectation when they are showing up. And that’s a thing which I keep telling them again and again. We sit down, and we have a look at the hand. And there are no obligations. And I think it is very very important. (P. No. 7.)

#### Addressing anxiety and perfectionism

When dealing with the psychological distress and negative emotions of their clients, participants use a rich variety of tools. Two participants were trained in psychotherapy and use their knowledge to help the musicians right after their diagnosis: “Usually I do this kind of short intervention. Psychotherapy intervention indeed” (P. No. 10).

Others studied body-oriented techniques, such as Alexander Technique or Dispokinesis,^1^ and since these approaches have strong cognitive elements, they prove to be appropriate tools to reach an ideal mindset in the patients. Some mentioned using Neuro-Linguistic Programming (NLP) or other frameworks, while musician-coaches primarily rely on their own experience and share the information which they found helpful in their own rehabilitation and in their practice. Each of these interventions is tailored to the specific case within the chosen modality.

Dealing with the musicians’ anxiety is one of the main objectives of different treatment strategies. As previous research has shown (Ioannou and Altenmüller, 2014), not all patients are suffering from clinical anxiety disorders, but given their situation, all affected musicians seem to have anxieties around their playing and rehabilitation. The importance of reducing anxiety was stressed by participant No. 6.: “it [anxiety] will modify the way they act, the way they do certain things, the attention they will put to the therapy, so it is absolutely important to reduce or solve that.” Also, understanding the source of the anxiety by asking the patients specific questions is often the first step toward supporting the musicians and helping the practitioners to create new strategies.

That anxiety is from: “I don’t know what to do, I don’t know how to do it, I’m lost.” And so, again, rather than looking at the anxiety […] I always prefer to look at the solutions. Why is that anxiety there, what is this person anxious about? What is going on in their lives? (P. No. 3.)

In serious cases, external treatment is suggested with a psychotherapist, but most practitioners aim to reduce their patients’ anxiety during the sessions by creating an accepting and open learning environment.

Perfectionism and the “need to control” were the next traits identified by the practitioners as harmful to the rehabilitation, and to address it, most often, reappraisal is suggested. One participant uses the following “mantra” to further this process: “I control the fact that I do not control” (P. No. 7). Others provided anecdotes of famous musicians making mistakes or dismantling the idea of perfection by providing a new framework with music as communication in the center, instead of avoiding making mistakes. Participant No. 3. Uses the expression “paint by numbers” to describe the desperate need to play every note flawlessly without considering the artistic content and explains it in the following way:

People assume that if you put all these notes in the right order, in the right way, it is going to be some work of art. But no matter how perfectly you place those notes, it will never turn out as a piece of art. Not without communicating something with it.

This approach is also aimed to reduce the anxiety around making mistakes and helps the client to refocus their efforts on their expressivity.

#### Rapport, shared personal experiences

In addition, all musician-coaches mentioned the importance of their personal story: sharing their own experience with the disorder builds trust in the musicians with MFD and gives hope that they can also overcome the condition. As one musician-coach articulated it:

I strongly believe that the fact that I got myself focal dystonia is an extremely strong point to them. As well as to me. So, they know I know. They know I can understand. They know that I can feel what they feel, not only physically, but also psychologically. Because I’ve been through it too. (P. No. 7.)

### Somatic approaches

#### Enhancing general body mechanics

Most practitioners agree that the problem is usually more widespread physically than the dystonic limb or facial muscles. Musicians with MFD generally carry a lot of muscular tension in their bodies and have inefficient postures both when playing and in everyday life, which—in the practitioners’ opinion—should be a therapeutic target in all practices. As participant No. 3. articulated: “It’s global before it’s specific. And that, I believe, is one of the biggest mistakes that educators make, that clinicians make, especially because of this specialization, that we all make.” These deficiencies are often undetectable for the musician with the disorder due to its habitual nature: “Some of my clients had never felt release. They have been walking around with so much tension for so long, that their body has learnt this, you know?” (P. No. 8).

To relieve these tensions from the entire body and reach a balanced and healthy alignment is one of the most important elements in many practices. Some use different body-oriented techniques, such as the Feldenkrais Method, Alexander Technique, or Dispokinesis in their practices to gain “healthy function” and a “global sense of release” (P. No. 13.) before they move on to the exercises targeting fine motor control.

#### Reducing the fear of the movement

The affected musician’s relationship to the instrument also needs to be addressed: the fear of the symptom and its unpredictable nature leaves many affected musicians feeling helpless or agitated which has a significant impact on how they approach the instrument and complete the prescribed exercises. Participants linked this fear to previously suffered traumas in connection to the instrument:

*…* when you first touch the instrument again, then this trauma situation comes back. When they open the case, everything reminds them of the situations they have experienced. That takes a long time to overcome. The fear of touching the instrument. (P. No. 14.)

To aid the process of overcoming the fear, practitioners also use sequential exercises, starting with very simple movements, and making each step attainable: “Every exercise is sequential […] And it is based upon successfully making a match between task and skill. And that builds a sense of self-esteem” (P. No. 3). Also, there is often a cognitive element added in the form of positive self-talk, which is continuously reinforced and repeated throughout the session:

It’s like a little devil sitting on your shoulder [the fear and anxiety], and it is talking into your ear. And if you recognise: oh, hello, it’s you, there you are (makes plucking noise, imitates flicking something away). You’re not necessary! (P. No. 14.)

#### Improving breathing mechanisms

Some practitioners also observe and correct the breathing patterns in their practices, not exclusively with embouchure dystonia patients. Breathing exercises are used to re-establish healthy breathing function, which is often agitated due to the anxiety and fear of approaching the instrument and can have further implications for the motor function as well:

So, someone has hand dystonia, and their hand is reacting, but I see them going like this (breaths in hyperventilation), I’m not going to talk about their hand. Because if I’m chronically hyperventilating, one of the symptoms is spasm. So, my hyperventilation might be leading to that tension. (P. No. 1).

The holistic approach also distracts the patients’ obsessive focus on the motor symptoms and leads to a broader sense of their body mechanics, i.e., embodiment. This cognitive element of the therapy can also be understood as a form of mindfulness and has additional benefits in reducing anxiety.

### Focus, habits, and nomenclature

#### Directing the attentional focus

All practitioners agreed that consciously directing the focus of attention is one of the most important elements of retraining; however, their opinions differed about the optimal direction of this focus. Some practitioners subscribed to the idea of encouraging external focus, due to its superiority to internal focus when performing already learned movements (Wulf, 2013). Channeling the focus of attention away from the affected body part can improve the motor symptoms but it is also aimed at reducing the fear of failure and the excessive need for control:

The most important is to solve this need for control in the patient […] when we are doing the neuro-rehabilitation exercises, we combine them with second activities. So, at the same time when the patient is doing a specific exercise on the instrument, we ask him to solve mathematical problems, coordinative exercises, games on the iPads et cetera. It is one of our strategies. (P. No. 4.)

However, others stated the opposite and prescribe increased focus on the affected body part and its motor movements in their practices and believe that it significantly furthers the rehabilitation: “I think the people who recovered fairly fast, they have trained themselves to be hyper-aware of what they are doing and how” (P. No.1). Among others, participant No. 6. felt that both types of attentional foci have value in terms of the rehabilitation:

Of course, the external focus is highly important. But I think we also need, as an additional tool, this internal focus, where we 100% focus on the forces, on the movements, like we just crawl into the hand, we are 100% in the hand, in order to go on, and step by step improve playing technique.

Also, as participant No. 10. pointed it out, the employment of different types must be carefully balanced depending on the situation: “internal focus when you are working on your tensions, and external focus when you are performing, this has to be taught. I think that’s important.”

#### Breaking habits and generating new experience

Interestingly, both types of instructions are aimed at “breaking the habit” of how the instrument is approached: “I try to break the habit of their approach first because whatever way they are used to approaching their instruments is kind of contaminated with dystonia” (P. No. 1). Moreover, many practitioners stated that emotions and the way the musicians express themselves through music are connected to the symptom; therefore, they actively dissect their clients’ motor movements from their musicality at the beginning of the retraining:

When you want to play in a very special way, you think that your emotions should feel very intense. And you increase and increase the tension, and you don’t know what you are doing is not necessary. I measured this for example with cellists – if they play in the high positions, they use the weight of the hand, and the fingertip gets up to 4kg on the string. (P. No. 14.)

And for most of my clients, I take the musicianship, and I put it on the shelf. And protect it. And I say: right now, your auditory signal has been connected to an abhorrent motor signal. So, what we have to do is just protect that for a bit, and we are just going to retrain healthy motion. And incrementally, so each step is successful. (P. No. 3.)

Building a new set of habits and re-learning certain movements is the core element of every retraining strategy, and it relies on the active participation of the musicians not only during but also between the sessions. Therefore, instead of following instructions, the musicians need to develop a sense of embodied and mental self-awareness to be able to observe their technique and practice, even when they are not under the direct supervision of the therapist. One successful strategy to achieve this is “provoking experience” and creating a space where musicians can exercise agency rather than prescribe an “ideal movement” they need to achieve. As participant No. 14. articulated: “I never say it is wrong or right. I always give the possibility to choose to the people. They can decide for themselves. I give them a situation where they can choose.”

The whole process of rehabilitation can last for years – it is a long and difficult process for most. To maintain the sense of being a musician, most patients are advised to restart performing in low-pressure situations with a carefully chosen literature as soon as possible. Some practitioners encourage them to discover and play within the limits of their ability, using improvisation.

#### Using carefully selected nomenclature

To enhance the process of rebuilding one’s instrumental technique, the nomenclature is also important; the affected musicians should not feel judged and have to keep an open mind to explore new possibilities in terms of movement, practice behavior, and instrumental technique. Participant No. 13. stressed that the words used to instruct the musicians make a “big difference,” especially when it comes to evaluating a motor movement; here, the focus needs to be on functionality and efficiency rather than meeting prescribed standards: “why would I think, that there is a right and a wrong way, vs. an efficient way or structurally functional way? So, our nomenclature makes a big difference.”

## Discussion

Rehabilitation from MFD is a very complex process, and to reach the primary goal – to eliminate involuntary movements and regain complete motor control when playing the instrument—practitioners use a wide variety of tools which influence cognition, emotions, and psychological states apart from working on the physical symptom. In some cases, these tools are clearly directed toward the patients’ mindset, but most often, they are intertwined with the exercises aimed at improving the affected motor movements. Many of the presented strategies have motor and cognitive-emotional contents at the same time; therefore, the grouping of these strategies is somewhat artificial. Nevertheless, the established framework provides a sense of structure that can be used as a precedent.

### Learning environment, psychological support, and rapport

The first group of presented tools are all aiming to establish a safe, calm, and supportive atmosphere, in which the musician can work more efficiently. The participants highlighted the necessity of actively and consciously constructing this environment due to the negative, even traumatic past experiences of their patients/clients and the accompanying psychological distress, and secondly, to mitigate the dismaying impact of the onset itself.

Previous negative experiences, especially if these take place in childhood, have been associated with various mental and physical health issues (Máté, 2011; Van der Kolk, 2014; Petruccelli et al., 2019; Zarse et al., 2019). Following up on this substantial literature, a recent initiative explored the childhood of musicians with MFD and found that compared to healthy samples, they tend to have more Adverse Childhood Experiences, especially emotional neglect during rearing (Alpheis et al., 2022), and potentially more psychological traumatization (Schneider et al., 2021). Similar life events emerged from the Grounded Theory study informing the present research (Détári et al., 2022) and the practitioners during this data collection recalled many musicians with MFD talking about traumatic experiences in their past (Détári and Egermann, 2022b). Additionally, the educational environment of those with MFD might have also been less than optimal: Détári et al. (2022) and Détári and Egermann (2022a,b) reported frequent occurrences of emotional abuse, socially prescribed perfectionism, and harmful teaching methods.

Additionally, it is important not to underestimate the psychological, emotional, psychosocial, and financial impact of the onset itself. Musicians spend decades honing their skills, often excluding other activities, and during this process, their identity intertwines with their ability to play their instruments (Kemp, 1996; Sanders, 1998). Thus, the onset disrupts not only their fine motor skills but also their embodied sense of self (Toner et al., 2016) raising serious doubts about their adequacy and personal value in society (Sparkes and Silvennoinen, 1999; Shilling, 2008). These influences often create serious psychological distress, anxiety about the future, intense emotional pain, self-blame, anger and frustration, and naturally, impatience toward oneself and the rehabilitation process.

The participants agreed that the distressing psychological states of the musicians in their practice create substantial barriers to rehabilitation. The motor output is extremely vulnerable to the psychological (Van Loon et al., 2001; Laursen et al., 2002) and emotional states (Juhan, 2003; Jansen, 2012) of the person executing them, and can have further implications for the capacity of learning. The increased levels of muscular tension in response to stressful and emotionally taxing stimuli and situations is a well-document fact (Larsson et al., 1995; Van Loon et al., 2001; Laursen et al., 2002); this tension can appear both in the postural (antigravitational) muscles (Larsson et al., 1995) that carry static loads or in muscles contributing to the movement itself (Laursen et al., 2002). The latter might also be a subconscious attempt to complete the task in a satisfactory manner: under psychological stress, limb stiffness (co-contraction of agonist and antagonist muscles) increases which reduces the degrees of freedom at the joints in order to stabilize the movement and gain better control over it (van Loon et al., 2001). Therefore, the musicians’ mental state cannot be separated from somatic learning and movement execution, and it is imperative to include it in the rehabilitation as a therapeutic target.

The participants used various tools to dismantle this barrier; firstly, the conscious use of holistic conversations with the musicians emerged from the data. Practitioners build rapport by touching on various topics in these conversations, including the musician’s concerns about their future or their recollections of their past. Narrating one’s story in order to “make sense” of one’s situation is an invaluable tool to support individuals with chronic illnesses (Jacobi and Mac Leod, 2011). By articulating the events leading up to the onset of the disorder and reflecting on and moving toward accepting the current situation, the musician might generate meaning and build resilience. McPherson et al. (2001) call this behavior “taking charge” over one’s situation, i.e., developing coping mechanisms to emotionally and physically adjust to the new reality. Moreover, listening to the musician’s stories and thoughts can be a source of meaningful information for the therapist (Jagosh et al., 2011; Gidman, 2013) given the high variability in the experiences, background, and displayed symptoms of the musicians with MFD; as Charon and Montello (2002) puts it: “the singular case emerges only in the act of narrating it and the duties are incurred by the act of hearing it” (p. 3). In other words, learning about the musicians’ life stories can inform the therapists of their psychological, psychosocial, and somatic needs.

The attributes of the environment in which the rehabilitation takes place, including the therapeutic relationship, seem crucial in terms of the success of the process and the musicians’ well-being. These findings align with decades of research examining the factors that positively contribute to various therapy outcomes; according to Lambert and Barley (2001), the therapist’s emphatic understanding, warmth, and ability to engage with the client are better predictors of a successful therapy than the specific therapy technique used. The construction of the interpersonal space, the established atmosphere and rapport, and the management of the expectations both of the musician and the person leading the session can have further implications for the outcome of the session. The practitioners aimed to consciously construct an environment which enables the musicians to have a more open-minded, curious, and relaxed attitude; they identified “safety” as the most important characteristic of the shared space. Establishing feelings of psychological safety might be challenging for a musician who has early adverse experiences; as Fang et al. (2014) observe, the levels of perceived safety are moderated by the individual’s experiences with rearing and can modify the social anxiety levels. Extrapolating from this, it is possible that task-specific negative experiences also influence how safe the person feels in the therapeutic environment: repeated exposure to unsafe situations during education or in professional settings in which the individual is ridiculed or humiliated can create fear of subsequent occurrences of the same psychological and emotional harm (Seligson and MacPhee, 2004; Edmondson and Lei, 2014). Given that the feeling of psychological safety is subjective and deeply personal, each musician might need a different approach to ensure their well-being during the retraining; however, establishing a welcoming, non-judgmental environment by displaying compassion is generally considered a good tool to enhance the feelings of safety (Latting, 1990; Wanless, 2016).

The practitioners also actively work with their patients’/clients’ anxiety and perfectionism. The used vocabulary when introducing a rehabilitation exercise alongside its goal and success criteria and when evaluating the musician’s performance was identified as an important tool for dismantling the client’s maladaptive mindset and behaviors. Practitioners stressed that observing the motor output and using it as a source of information rather than imposing expectations on oneself is a cognitive strategy which can be highly effective in loosening perfectionist tendencies. This leads to a dialog-based debriefing after each trial; a method Patston (2014) suggests for music educators to avoid inducing unrealistic expectations and increase student engagement. This constructivist approach is further justified by the wide variety of both motor and non-motor symptoms in musicians with MFD. Constructivism in education rejects the notion of absolute truth as an objective entity (Webster, 2011) and sees it as the result of the student’s interactions with and responses to the presented material. In other words, knowledge is constructed as part of social activity (Shively, 2015); therefore, it is unique for each learner. Using this notion in MFD retraining means that the musician’s development is created through constant communication with the therapist and is fundamentally based on their individual symptomatology and interactions with the given instructions and exercises, such as their physical sensations, emotional and psychological states, and currently attainable capabilities.

Furthermore, when practitioners aim to extrinsically motivate the musician, the content and quality of the praise might also influence the musician’s mental state. Accomplishment-based praise might reinforce the rigid and perfectionistic mindset, while effort-based praise can further motivation (Dweck, 2000; Dweck and Master, 2012). Given that the musician’s ability is impacted by the MFD symptoms, and it is not under conscious control, honoring their efforts toward the goal rather than evaluating the output is much more realistic and could enhance self-efficacy and motivation.

### Somatic approaches

The biomechanics of instrumental technique is one of the obvious targets of the rehabilitation; however, the participants highlighted the importance of thinking about movement in a broader and more holistic way. Firstly, many of them emphasized that apart from the affected fine motor skills, the overall body mechanics and posture also need careful attention. This aligns with several documented treatment approaches, for example, both Tubiana (2003) and Ackermann and Altenmüller (2021) stressed the importance of including larger structures (e.g., torso, scapula, spine) in the rehabilitation, while other documented rehabilitation protocols use general fitness exercises as part of the treatment (Byl et al., 2009).

Attending to the movement behavior not just on a mechanical level but taking the musicians’ previous experiences into account must also be stressed: the rehabilitation of the motor function is inseparable from the reappraisal of the accompanying emotions. Many participants acknowledged that fear and unconscious avoidance appear in the affected musicians when they attempt any performance-related movements. Bennett et al. (2016) observed similar tendencies in athletes experiencing the “yips” and Lost Movement Syndrome (LMS) as they highlighted the “feelings of fear associated with the affected movement pattern, both in relation to the consequence of experiencing movement breakdown, and to avoidance of executing affected moves” (p. 65). This can be understood as anticipatory anxiety which can lead to a heightened autonomic response, decreases blood flow to the prefrontal cortex (Simpson Jr et al., 2001), and raises respiratory frequency (Masaoka and Homma, 2001), especially in individuals with high trait anxiety. It seems obvious that these reactions can have an adverse effect on the learning process itself and point toward the importance of structuring the rehabilitation strategy to eliminate their negative impact. The most frequently used therapeutic response to this fear in the sample was to start with very simple and attainable movements and gradually increase the difficulty, while carefully monitoring the musicians’ physical and emotional responses.

It also needs to be mentioned that musician-coaches also attended to the biomechanical quality of the fine motor movements associated with playing the instrument. Opposing the general but unexamined notion is that before the onset, the movement patterns of the affected musicians were optimal, the musician-coaches agreed that many of their clients had ineffective and unhealthy technique even before the onset. Data obtained from affected musicians (Détári et al., 2022) show similar tendencies: many of the participants in the sample struggled with certain technical aspects of their playing, often for years before the onset, sometimes even through their entire career. Even more interestingly, those who shared these experiences reported that their first symptoms appeared when attempting to perfect said skill. A similar tendency was found in Lost Movement Syndrome: Day et al. (2006), when interviewing elite trampolinists affected by this task-specific movement disorder, traced the onset back to shortcomings in the initial movement acquisition. This characteristic of the instrumental technique preceding the onset signals that during the retraining, instead of rehabilitating the old movement patterns, some adjustments and corrections need to be made. Therefore, even if the therapy is not led by a musician-coach, the support of a music educator might be necessary; as Tubiana (2003) writes, “the collaboration of instrumental teachers is also desirable and may be of great help” (p. 169).

The idea of inefficient playing technique as an origin is also supported by Sadnicka et al. (2018). According to their recently suggested framework, the symptoms are compensatory movements of an otherwise healthy motor system rather than unexplained reactions of an incapacitated neural network (Sadnicka et al., 2,108). In other words, the employment of a suboptimal movement pattern can lead to a misalignment between the increasing requirements of the musical tasks as the career of the musician advances and their ability to meet them.

### Focus, habits, and nomenclature

Practitioners also acknowledged the fact that the way patients think, focus, and direct their movements cognitively can create significant changes in the quality of the motor output. Some of the participants arrived at this conclusion intuitively or deduced it from their personal experience, but it is also supported by the literature. As an example, it has been shown in the field of sports psychology that modifying the attentional focus can lead to changes in muscle tone, consistency, accuracy, endurance, and even the degrees of freedom in joints (for comprehensive reviews, see Wulf, 2013 and Wulf and Lewthwaite, 2016). Sadnicka et al. (2018) make a similar point, specifically about task-specific dystonias: “the influence that misdirected cognitive influences can have on skill performance is worth emphasizing” (p. 123).

While sports psychology literature firmly established the superiority of external focus when acquiring or performing various skills (Wulf and Lewthwaite, 2016), the majority of the studies explored the role of attentional focus in the movement of healthy individuals (see Wulf, 2013). Very little is known about attentional focus in the context of movement disorders (Hunt et al., 2017), such as MFD, especially regarding their treatment. There is some evidence that consciously directing the attentional focus externally has its therapeutic uses when attempting to enhance movement behavior: a growing number of articles discuss the positive impact of external focus on Parkinson’s patients’ postural sway (Wulf et al., 2009; Jazaeri et al., 2018), and stroke patients’ “functional reach” (Fasoli et al., 2002). Hunt et al. (2017) also argue that the neurocognitive aspect of motor learning should be fully exploited by physiotherapists when attending to patients with musculoskeletal and movement disorders to achieve better treatment outcomes.

Instructing musicians to direct their attentional focus differently can be easily achieved; however, there were differing opinions about the optimal focus point among the participants. Some wholeheartedly prescribed to the idea of encouraging external focus during the rehabilitation by citing the literature or using their personal experience to inform their strategy, while others highlighted the importance of developing internal focus to supervise the corrections in the performance-specific movements. Other practitioners argued that both types of attentional focus need to be exploited to achieve better movement control.

The solution to this contradiction could be to abandon the binary view of attentional focus (i.e., external vs. internal): as Allingham et al. (2021) argue, the simplicity of this understanding might not be appropriate to fully capture the complex procedure associated with playing an instrument: additional factors, such as the auditory feedback, musicality, emotions, and the felt resonations of the created sounds might complicate this equation. Therefore, they introduced an additional focus condition into their study on violin bowing (Allingham et al., 2021): a somatic focus, targeting the sensations resulting from the interaction with the instrument (i.e., resonance) within the body, and found that this condition was superior to both internal and external conditions in terms of movement effectiveness. A memoir of a recovered trombonist (Chopyk, 2021) reinforces the benefits of this “somatic” focus: they achieved the best rehabilitation results by turning their attention toward the air resonating inside their wind instrument. It seems that consciously directed attentional focus plays a significant role in the rehabilitation process, but apart from the qualitative data obtained by this study, there is no literature available on the role of attentional focus in the rehabilitation of MFD. The topic, however, is worthy of attention and further, detailed exploration.

Breaking the habitual, and symptom-triggering way of approaching the instrument and generating new experiences was also reported as an important tool to rehabilitate musicians with MFD. As it was stated by the participants, the habitual instrumental technique carries not only the involuntary and uncontrollable movement patterns of the disorder, but also evokes habitual posture and breathing patterns and accompanying negative emotive states. This is dubbed in the literature as “implicit body memory” (Fuchs, 2003; Jansen, 2012), and explained by Juhan (2003) in the following way: “the feeling states that are being experienced at the same time learning is taking place are recorded along with the performance or the information, and closely associated with future recall” (p. 372). Byl et al. (2000) make a similar observation about the movements of task-specific dystonia sufferers when they return to performing the triggering task: “the hand remembers something about the target task” (pp. 289–290). Therefore, to avoid reinforcing the symptom, the habitual approach needs to be altered in some way.

The therapeutic responses to these issues varied among the participants. Some suggested introducing a new routine when engaging with any playing-related behavior, such as adjusting postural alignment, breathing exercises, or simply including distractions to change the attentional focus. Many musician-coaches stressed the importance of making each task simple, attainable, and moving forward incrementally to ensure that the musician gains trust in their ability to perform the exercise. Essentially, this approach points toward the development of self-efficacy in the musician, i.e., the “belief in one’s ability to accomplish a specific task” (Hendricks, 2015, p. 32). Self-efficacy has been discussed by many researchers in the field of music education and performance (McCormick and McPherson, 2003; McPherson and McCormick, 2006; Hendricks, 2014) and the main findings show that the musician’s level of self-efficacy is a better predictor of achievement that their actual skillset.

One of the cornerstones of self-efficacy is generating mastery experiences (Zelenak, 2010; Hendricks, 2015) which might be challenging when attending to musicians with MFD; the experienced symptoms clearly undermine their sense of accomplishment. As Chopyk (2021) articulates: “the mind is in disbelief that even the most basic function can be accomplished. The player simply cannot imagine what it would be like to successfully play even the easiest things.” Therefore, setting obtainable goals, however simple these might be, can support the musician to increase their confidence through accomplishing successful trials and, at the same time, avoid anticipatory anxiety. For the majority of the musicians with MFD who experience task-specific symptoms (Hofmann et al., 2015) the body’s capability to move in a controlled way in the absence of the instrument can be exploited in creating the exercises. Examples of this might be simple breathing exercises while touching the mouthpiece of a wind instrument to one’s face without the attempt to make an embouchure or touching the piano keys with the back of the hand instead of the fingers. These accessible tasks might restore a sense of control and trust in one’s own body and its ability to move in the presence of the instrument without experiencing symptoms. However, the simplicity of these types of exercises might carry a potential adverse effect as well: one’s subjective perception of the achievement moderates the sense of self-efficacy (Bandura, 1997) and if the musician compares the completed task to their pre-onset ability, the evaluation will surely be negative. Therefore, an important part of the provided guidance is to communicate the exercise’s role and place in the long-term rehabilitation strategy by using carefully chosen nomenclature to help the musician to reappraise their performance.

### Outlook

Until recently, the diagnosis and treatment of MFD were exclusively the tasks of neurologists. However, newly emerging frameworks of understanding MFD, including non-motor symptoms (Altenmüller et al., 2014) and possibly, very specific technical issues of instrumental playing (Détári et al., 2022; Détári and Egermann, 2022a,b) requires music educators and musicians to participate in the process. As seen in this study, some musician-coaches specialize in providing therapeutic instrumental sessions; however, music educators can also help to develop and provide preventative educational tools.

The evidence (Détári et al., 2022; Détári and Egermann, 2022b) of the link between the quality of educational experiences and the onset of MFD can be used to inform preventative strategies. Based on the findings, the three main pillars can be outlined: (1) creating an accepting and safe working environment for the student, (2) being informed about the biomechanics of the technique and actively monitoring if the student is playing in an efficient and healthy way (3) withstand the pressure from the music industry which promotes, encourages, and celebrates “perfect” and hugely demanding performances, often at a very young age. The last point is hugely important for developing a stable and well-supported technique, giving the students authority over their own learning, and avoiding psychological pressure. The main goal should be to support the development of a healthy technique which requires strong foundations. As one of the participants put it: “Young children need the best teachers” (P. No. 10).

Music educators are also ideally positioned to detect the first signs of movement deterioration. While the onset of the condition typically happens in the 4^th^ decade of the playing career, in study samples, participants as young as 17 are not rare (Conti et al., 2008). In cases where the developed technical issues in a young musician appear unsolvable by traditional educational means, with a basic awareness and understanding of the condition, music teachers can direct their students toward appropriate support. With this approach, affected musicians can receive help at the earliest signs of deterioration, which gives them a better chance to recover. Therefore, raising awareness among music educators could be a helpful tool to support recovery.

When it comes to treatment, especially behavioral therapies, the role of music educators can be even greater. Since they have in-depth knowledge of the instrumental technique, i.e., the motor patterns which need to be recovered, their expertise is crucial in developing the appropriate retraining programs and practice schedules. They also have personal experiences of the schedule, lifestyle, unique external influences, and stressors and pressures which are part of the profession; therefore, they can approach the affected musicians in a compassionate and understanding manner. The knowledge of musicians who recovered from the condition is especially invaluable: they are highly motivated to provide better support than they received when they were recovering from the condition and their personal experience of the process of recovery and their developed methods can offer important insights. At the same time—as the example of the six musician-coaches who refused to take part in the research—they might be overly attached to their personal method, excluding other potentially valuable resources.

In summary, more research is needed to explore the tools used in private practices with participants who are willing to collaborate. As a first step and following the findings of this research, further studies are planned to gather data about the clients’/patients’ perceptions of the received treatment, particularly their experiences with the strategies aimed at non-motor symptoms.

### Limitations

In this study, practitioners self-reported their therapeutic strategies for supporting musicians with MFD which carries potential bias, and due to the nature of their practices, the outcomes of treatments were not systematically documented. Nevertheless, the participants spent a significant time developing and enhancing these tools and worked with a substantial pool of patients, suggesting unique expertise in the field.

## Conclusion

MFD is a multifaceted condition with a wide range of motor and non-motor symptoms, which, apart from the genetic predisposition and maladaptive neurological changes (Altenmüller, 2003; Byl, 2006; Altenmüller and Jabusch, 2010; Lohmann et al., 2014) at least partially originates from personality traits, maladaptive cognitive strategies, socio-environmental factors, and learned behaviors (Schneider et al., 2021; Alpheis et al., 2022; Détári et al., 2022; Détári and Egermann, 2022a,b). Practitioners are not only aware of the impact of these factors on the rehabilitation, but they also developed certain strategies to address them. As the data shows, there is a wide variety of tools they employ, but we know very little about their efficiency. The literature assessing behavioral therapies for the condition exclusively discusses the treatment of the physical symptoms, even though the influence of various psychological and behavioral factors appears to be significant.

There are many ongoing successful practices led by former sufferers, somatic practitioners, and medical professionals, with decades of experience which have never been researched or assessed; thus, the accumulated knowledge of these individuals remains largely unexploited and benefits only the clients they directly work with. More research is necessary to understand the protocols of these practices, especially the tools they use to meet the psychological and behavioral needs of musicians with MFD. In order to enhance MFD treatment, it is necessary to establish communications between practitioners from various backgrounds and experiences; as one of the musician-coaches put it (No. 3.): “this needs to be a collaborative effort between us, psychologists, physiologists, the neurologists, the actual musicians, and music educators.” The result of this collaboration should be a clear consensus on how the condition should be addressed and how the therapy can be aided by psychological and behavioral tools.

## Data availability statement

The raw data supporting the conclusions of this article will be made available by the authors, without undue reservation.

## Ethics statement

The studies involving human participants were reviewed and approved by Arts and Humanities Ethics Committee of the University of York. The patients/participants provided their written informed consent to participate in this study.

## Author contributions

The author confirms being the sole contributor of this work and has approved it for publication.

## Conflict of interest

The author declares that the research was conducted in the absence of any commercial or financial relationships that could be construed as a potential conflict of interest.

## Publisher’s note

All claims expressed in this article are solely those of the authors and do not necessarily represent those of their affiliated organizations, or those of the publisher, the editors and the reviewers. Any product that may be evaluated in this article, or claim that may be made by its manufacturer, is not guaranteed or endorsed by the publisher.

## Figures and Tables

**Table:** 

Domain	Questions
Introduction + ethics	Greeting, permission to record the interview, summary of the ethics, rapport
General information about the participant’s practice	How long have you been treating people with MFD? How did you start working with MFD sufferers? Roughly how many people do you treat per year? How long does the rehabilitation last on average? How often do you see your clients/patients? How much time do you spend with them?
Non-motor symptoms	Do you notice salient psychological characteristics in the sufferers? How would you describe them? Did you notice similarities in their professional behaviour? Do your clients/patients share details about their personal/professional experience? If yes, are there any similarities between these recollections? Are you aware if your clients suffered any kind of psychological trauma before the onset? Are your clients/patients asked about health-related behaviour? If yes, are there frequently reported issues? Are there any other characteristics musicians with MFD share in your opinion? Follow-up questions about the topics in the framework which were not spontaneously mentioned.
The issues’ effect on the rehabilitation	What are the most important factors influencing the retraining? Subsequent questions based on the answers from the previous section: Do you think that the personality traits you mentioned influence the treatment? Do you think that the behaviours you mentioned can enhance or hinder the process?
Addressing non-motor symptoms	Do you think that the [non-motor issues mentioned by the practitioner] can be addressed as part of the treatment? Do have any specific strategies to address the [non-motor issues mentioned by the practitioner]? Are there other types of therapy you suggest to them to complement the treatment?
Summary, closing the interview, ethics repeated	Asking for any last thoughts, repeated ethics, closing the interview. Thanking the participant for taking part in the research.

## Footnotes

^1^Dispokinesis is a form of movement training and therapy, developed specifically for musicians and stage artists by Gerrit Onne van de Klashorst at the University of Amsterdam.
